# Time-Based Formulation Strategies for Colon Drug Delivery

**DOI:** 10.3390/pharmaceutics14122762

**Published:** 2022-12-09

**Authors:** Andrea Gazzaniga, Saliha Moutaharrik, Ilaria Filippin, Anastasia Foppoli, Luca Palugan, Alessandra Maroni, Matteo Cerea

**Affiliations:** Sezione di Tecnologia e Legislazione Farmaceutiche “Maria Edvige Sangalli”, GazzaLaB, Dipartimento di Scienze Farmaceutiche, Università degli Studi di Milano, Via G. Colombo 71, 20133 Milano, Italy

**Keywords:** colon targeting, time-controlled release, pulsatile release, time-dependent release, small intestinal transit time, in vivo human data, *γ*-scintigraphy

## Abstract

Despite poor absorption properties, delivery to the colon of bioactive compounds administered by the oral route has become a focus of pharmaceutical research over the last few decades. In particular, the high prevalence of Inflammatory Bowel Disease has driven interest because of the need for improved pharmacological treatments, which may provide high local drug concentrations and low systemic exposure. Colonic release has also been explored to deliver orally biologics having gut stability and permeability issues. For colon delivery, various technologies have been proposed, among which time-dependent systems rely on relatively constant small intestine transit time. Drug delivery platforms exploiting this physiological feature provide a lag time programmed to cover the entire small intestine transit and control the onset of release. Functional polymer coatings or capsule plugs are mainly used for this purpose, working through different mechanisms, such as swelling, dissolution/erosion, rupturing and/or increasing permeability, all activated by aqueous fluids. In addition, enteric coating is generally required to protect time-controlled formulations during their stay in the stomach and rule out the influence of variable gastric emptying. In this review, the rationale and main delivery technologies for oral colon delivery based on the time-dependent strategy are presented and discussed.

## 1. Introduction

For more than three decades, release of drugs to the colon has been an important research topic in the area of oral delivery and formulation [1,2]. Interest in this particular field was formerly sparked by the need for targeted and more effective treatment of Inflammatory Bowel Disease (IBD), including ulcerative colitis and Crohn’s disease [3,4]. Since then, other pathologies of the distal intestine, such as irritable bowel syndrome, infectious diarrhea, diverticulitis and dysbiosis, have become targets for colonic delivery. 

While local therapeutic applications have steadily been pursued until the present day, a range of different goals have emerged over the years. Notably, following the biotech revolution, colonic release has been harnessed to reach increased peptide bioavailability via non-invasive and patient-convenient oral administration, mainly because of less abundant digestive proteases in the large than in the small intestine [5,6]. In a particular instance, delivery of β-lactamases to the colon has been proposed to degrade unabsorbed antibiotic residues, thus limiting the spread of resistant bacterial strains [7]. More recently, targeting the colon with antigens, formulated with the aid of nanotechnologies to promote mucosal uptake, has been explored as a possible strategy for intestinal vaccination [8].

Until the mid-1980s, colonic delivery was primarily pursued to treat IBD, and at that time, sulfasalazine was the drug of choice even though it had already been shown that 5-aminosalicylic acid (5-ASA) was the active moiety. To our knowledge, the first talk on colonic drug delivery at an international meeting was given as an invited lecture at the 17th Annual Symposium of the Controlled Release Society (CRS) held in Reno, NV, in July 1990 [9]. It is interesting to note that the opening of the talk was: “The cliché there is nothing under the sun applies to colonic drug delivery. Primitive men found and utilized several plant sources that act being delivered to the colon. The anthraquinone glycosides in Cascara and Senna are examples”. The cliché referred to the microbiological approach, which in those years was the only strategy recognized for oral colon targeting.

The research was in fact mainly focused on polymeric prodrugs or polymers, intended to be degraded selectively in the colon by the action of enzymes, namely azoreductases or glycosidases, produced by different species of bacteria present in that region only. The prodrugs, as well as some polymers that were used alternatively as coating or matrix-forming agents, were obtained by chemical synthesis and suffered from regulatory problems being considered New Chemical Entities (NCEs) [1,10,11,12]. Later on, the use of mixtures of natural polysaccharides with insoluble polymers was proved to be promising [13,14].

**Figure 1:**
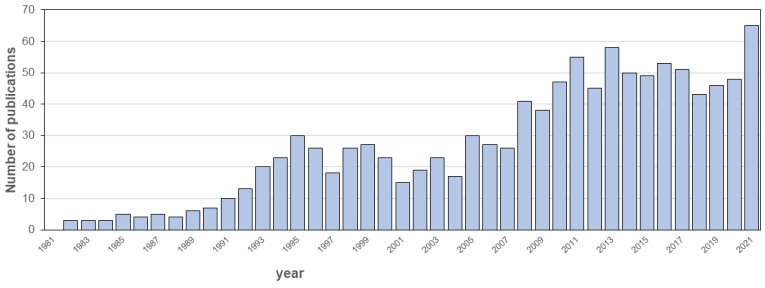
Publications on targeted colonic release in 1981–2021. Source Scopus.

The number of articles on targeted colonic release, mainly referring to substrates selectively degraded by the microbiota, has progressively increased over the years, at least until the late 90s (Figure 1). For 2022, based on current data, a similar number of papers to that of 2021 is expected.

Actually, a number of papers based on different approaches date back to the early 80’s. S.N. Rasmussen and coworkers developed a slow-release pellet formulation of 5-ASA (Pentasa^®^, Ferring Pharmaceuticals, København, Denmark) to treat IBD, including Crohn’s disease that also affects the small intestine, avoiding most sulfasalazine-related adverse reactions [15,16].

Specific colon delivery of sulfasalazine and 5-ASA was first attempted by M.J. Dew and colleagues by the use of enteric-coated formulations based on Eudragit^®^ S, a pH-dependent polymer soluble at pH > 7 [17,18,19]. Such formulations were designed relying on the hypothesis that the pH increases gradually from the stomach to the colon. This assumption subsequently proved to be inconsistent, as the pH was demonstrated to rise to values above 7 in the ileum, and a sharp drop to about 6.4 in the cecum was highlighted, followed by a slow aboral rise [20].

Nevertheless, a number of products based on the pH-dependent approach are currently on the market [21]. In this regard, pharmaceutical companies confirm the tendency to be rather conservative. Recently, different attempts have been reported to improve pH-controlled colon delivery platforms [21,22,23,24,25]. The Phloral^®^ technology, based on a combination of pH- and microbiota-sensitive approaches, represents a major step forward and, a couple of years ago, led to the launch on the market of a drug product containing 1.6 g of 5-ASA [24,26].

In subsequent years, along with the possibility of pH monitoring, the main inputs to a novel design of colonic delivery systems came from studies of gastrointestinal (GI) transit using *γ*-scintigraphy. In a review article published in 1985 some “illuminating” passages can be found that first indicated a new strategy for oral colon delivery: “The relative constancy of the transit of delivery systems in the small intestine can be exploited for the design of systems that will provide positioned release…” and “…the constancy of the intestinal transit time (3 ± 1 h, mean ± SD) as a means of delivering drugs specifically to the colon” [27].

This concept came from a meta-analysis concerning the transit times in the stomach and small intestine of different dosage forms, both in fed and fasted condition, assessed by *γ*-scintigraphy. The data originated from more than 150 healthy subjects. Small Intestinal Transit Time (SITT) was calculated and reported as the difference between colon arrival and gastric emptying, including possible stagnation at the ileo-cecal junction [28].

**Figure 2:**
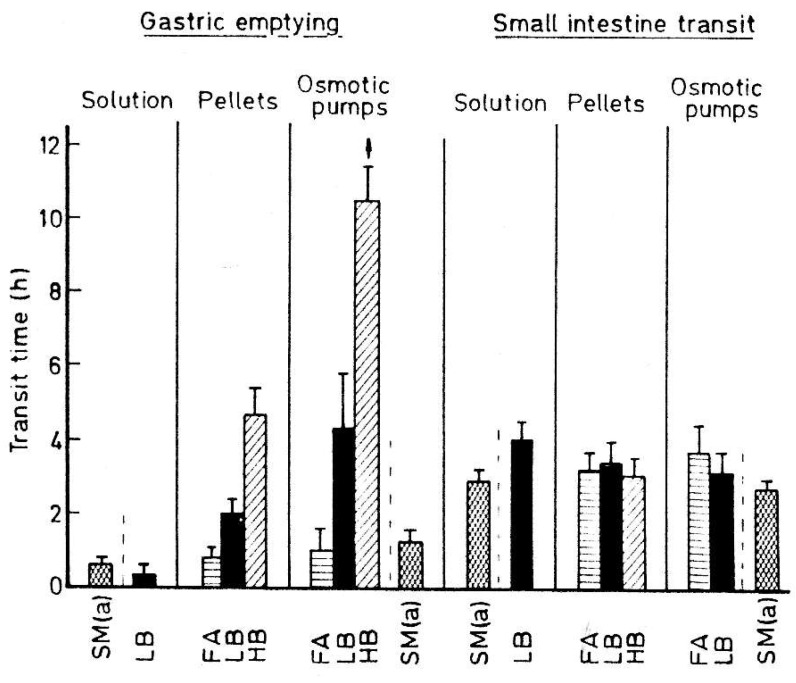
GI transit of dosage forms. Reprinted with permission from Ref. [27]. 1985, Elsevier. LB: Light Breakfast (1500 kJ); HB: Heavy Breakfast (3600 kJ); FA: Fasted; SM: Standard Meal (solution and solid fiber 1–5 mm in length).

In contrast to highly variable stomach residence, SITT for non-disintegrating dosage forms was practically independent of the dosage form characteristics and fed/fasted conditions of the subjects (Figure 2) [27,28,29,30].

**Figure 3:**
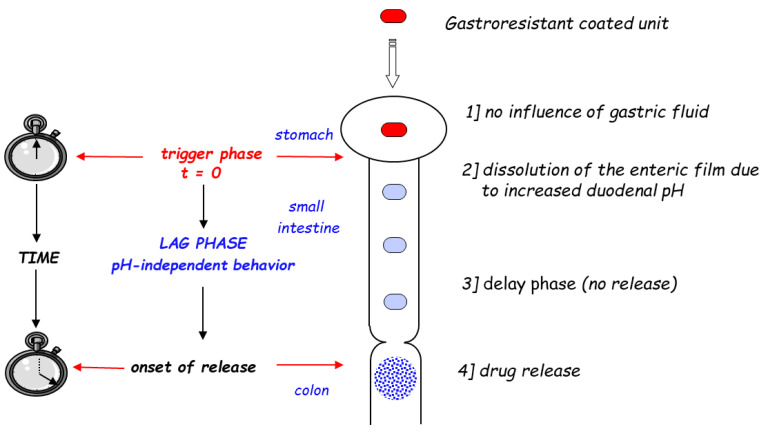
Schematic representation of time-dependent systems for colon drug delivery.

The basic architecture of the systems relying on the novel strategy to target the colon was defined as follows: “…The delivery system can be coated with an enteric coating material (cellulose acetate phthalate or methacrylate) which dissolves when the delivery system enters the duodenum. A second coating then provides the delayed release property so that the system starts to deliver drug by the time it reaches the colon” [27]. Following Bob Davis’s insight, a number of dosage forms were proposed defined as “time-controlled”, “time-dependent” or “time-based” colon drug delivery systems. The diagram in Figure 3 illustrates in more detail the design principle behind this strategy.

Following oral administration, the unit is expected to remain intact in the stomach, where the residence time is unpredictable, being protected by an external gastroresistant polymer coating [31]. Upon entering the small intestine, it would “know”, thanks to the pH change, that it has left the stomach, i.e., the triggering phase. Once in the duodenum the enteric coating dissolves, and the delay phase (during which no release should occur) can start, lasting at least 4 h (time supposed to be required to reach the colon). The delay phase relies on a solvent-activated mechanism, such as dissolution, erosion, dispersion or swelling of different polymeric or non-polymeric components.

Various delivery technologies have been followed to this end, based on progressive erosion or rupture of hydrophobic coatings, or on slow swelling/dissolution/erosion of hydrophilic coatings, of capsule plugs or of capsule shells. After the delay phase, the release takes place according to the design and the characteristics of the drug-containing core (immediate or prolonged release). In principle, all the pulsatile, also defined as “delayed”, release systems provided with an outer gastroresistant film would be eligible for colon targeting by the time-dependent approach.

Only delivery systems that have undergone human *γ*-scintigraphy studies, the results of which are published in the literature, are here reviewed and discussed. So far, few colon delivery platforms have been investigated in humans, namely the Chronotopic^®^, Pulsincap^®^, Time Clock^®^, Colon-Targeted Delivery Capsule (CTDC) and Egalet^®^ (Table 1).

All formulation strategies intended for colon delivery suffer from some limitations, related to the poor reproducibility of physiological properties (pH, bacterial population, transit time) and the possible impact of pathological conditions, namely ulcerative colitis and Crohn’s disease.

A major criticism addressed to the time-dependent approach concerns the reproducibility of SITT. However, it should be noted that in all published human studies, about 100 cases reported, time-based systems have largely shown their ability to target the colon.

Since the mid-1980s, much research work has been done for at least three decades to gain more insight into GI transit. The advent of imaging techniques, particularly *γ*-scintigraphy, and also Magnetic Marker Monitoring (MMM), as well as more easily accessible indirect methods, such as the hydrogen breath test and use of marker drugs, allowed a gain of decisive knowledge for the design and development of oral colonic delivery [37].

**Figure 4:**
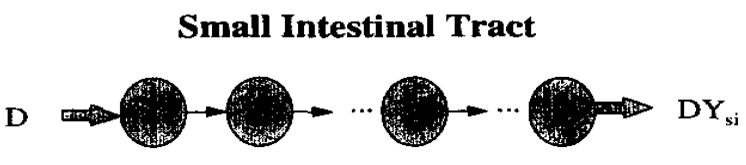
A schematic diagram of compartmental model with linear transfer kinetics. Reprinted with permission from Ref. [38]. 1996, Elsevier.

**Figure 5:**
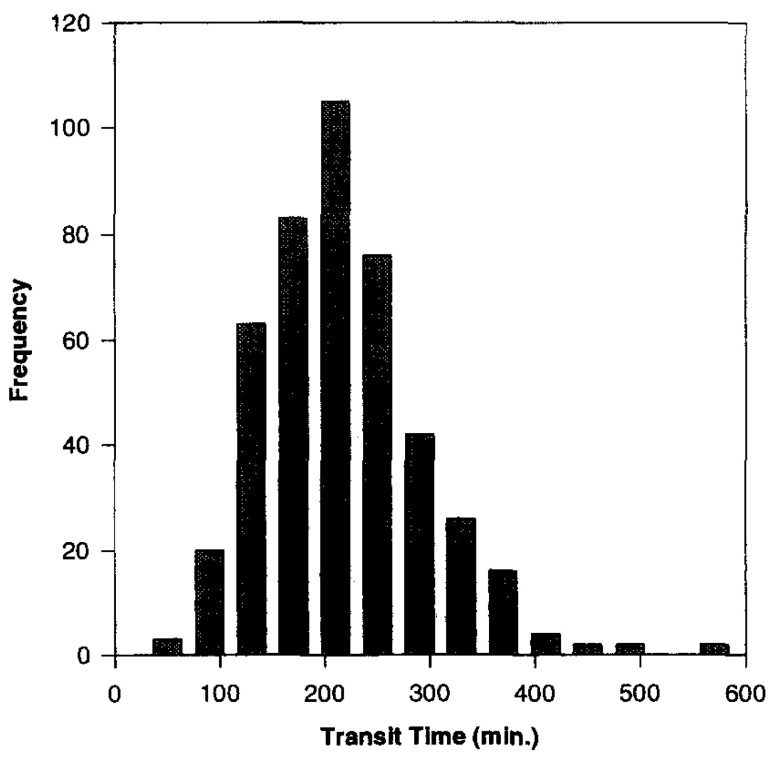
Small intestinal transit time distribution based on the frequency. Reprinted with permission from Ref. [38] 1996, Elsevier.

**Figure 6:**
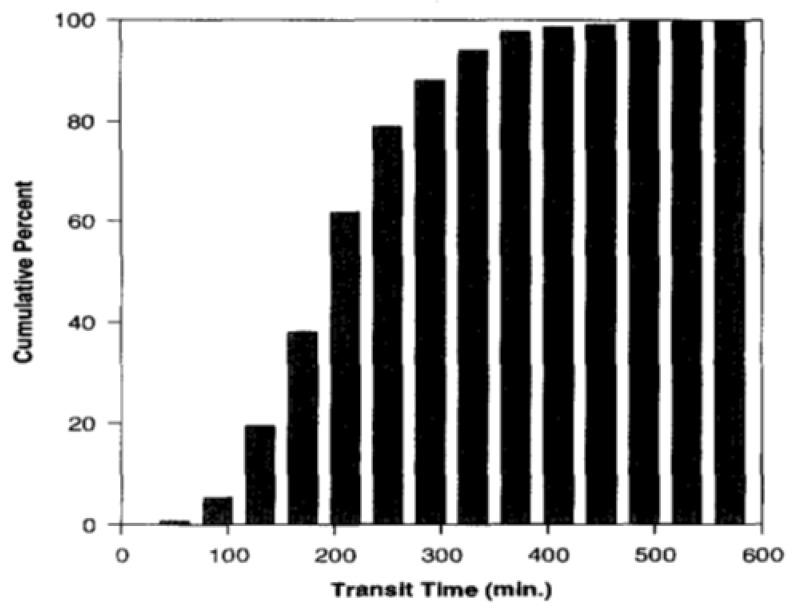
Cumulative percent graph of the small intestinal transit time based on its distribution. Reprinted with permission from Ref. [38]. 1996, Elsevier.

**Figure 7:**
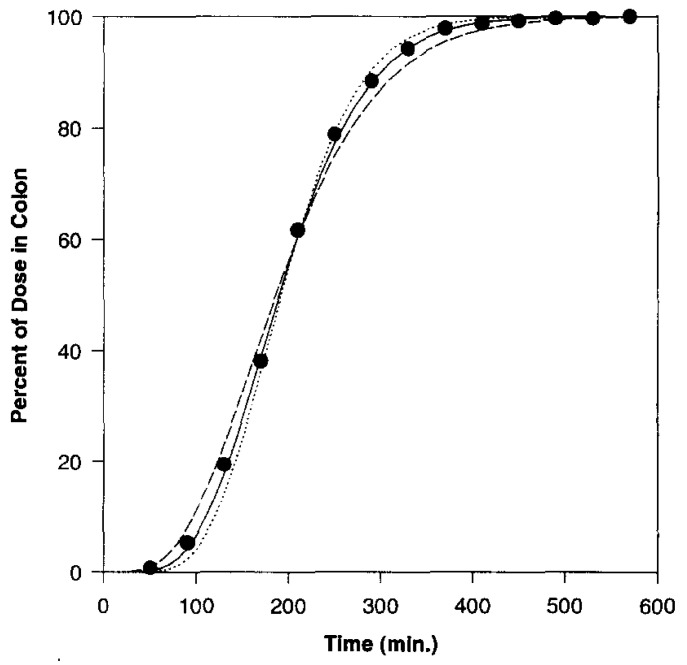
Estimate of human small intestinal transit flow using compartmental models. Reprinted with permission from Ref. [38]. 1996, Elsevier. _ _ _ five compartments; ____ seven compartments; …… nine compartments; ● ● ● cumulative percentage.

In 1996, a new meta-analysis was published that included a total of more than 400 SITT data from human subjects [38]. From these, the median, mean, standard deviation and 95% confidence interval were calculated, which were 191, 199, 78 and 7 min, respectively. Thus, a cumulative percentage graph was created and then used to build the N compartmental transit model (schematic diagram with linear transfer kinetics in Figure 4). Such a model consists of N compartment accounting for transit flow in the small intestinal tract. Each compartment has the same transit time but may have a different volume and flow rate. From this analysis it was assessed that SITT is very well described by a seven-compartment model. Indeed, the predicted cumulative SITT distribution was practically superimposed on the experimental data (Figure 5, Figure 6 and Figure 7).

A comprehensive review was performed by K.H. Yuen, reporting the mean SITT to be in a 3–4 h range, closely similar to that of food and water [39]. No influence of the physical state of the dosage form nor of the presence of food was highlighted. Regarding the timing of a meal, according to H.M. Fadda et al., SITT can significantly decrease if the food intake occurs when the dosage form is located in the duodenum, possibly due to an increased peristaltic activity [40]. However, in agreement with other studies, SITT was not affected by fed or fasted conditions [40,41]. It was concluded that the mean SITT is quite consistent among dosage forms and studies, while individual values can vary widely.

**Figure 8:**
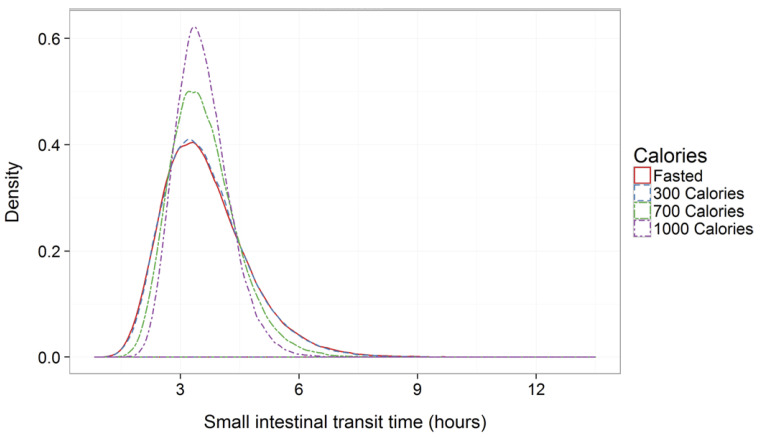
Meta-distribution density plot of gastrointestinal transit times in the small intestine. Reprinted with permission from Ref. [43]. 2016, Springer Nature.

Food effects were further evaluated by a quantitative meta-analysis to estimate SITT both in the case of non-disintegrating single-unit (tablets) and multiple-unit (pellets/multi-unit tablets) solid dosage forms [42,43]. In particular, 29 studies were involved, and 125 means and standard deviations were included in the meta-analysis, which focused on the influence of meals with different caloric content on SITT (Figure 8). This analysis showed that while the meta-average of SITT was unaffected by prandial status or type of dosage form, the meta-variability (SD) of SITT was significantly reduced as the caloric content of the meal increased.

**Figure 9:**
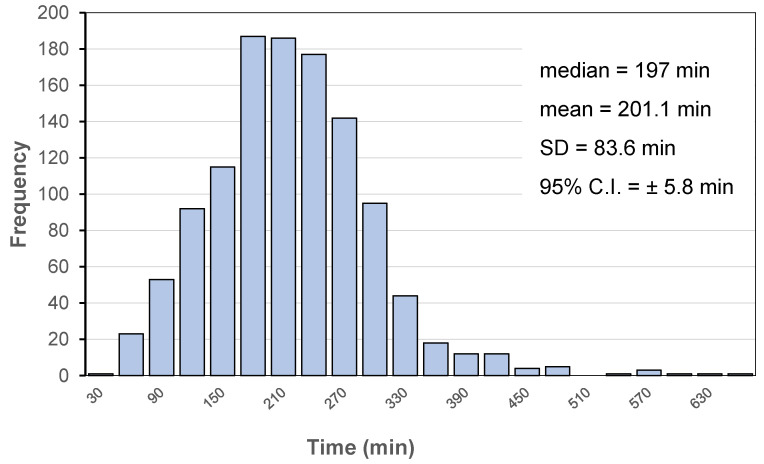
SITT distribution in 1179 volunteers. Data collected regardless of dosage form type and food intake, organized into 22 classes each of 30 min.

Recently, our group has collected a total of 1179 individual SITT data, resulting in median, mean, SD and 95% confidence interval of 197.0, 201.1, 83.6 and 5.8 min, respectively (Figure 9). Interestingly, only 5% of the reported SITTs were longer than 330 min.

The colon release strategy based on the exploitation of transit time in the small intestine is generally highly criticized, citing its poor reproducibility in both pathological and physiological conditions. Although some statistical concerns remain, the analysis of a large amount of experimental data collected from hundreds of volunteers indicates a very solid estimate of the mean. The most evident variability is sustained by a relatively small number of subjects showing SITT longer than 6 h. By designing delivery systems with delay times after gastric emptying that are more extended (5–6 h) than the average SITT value (+2 or 3 h), the risk of drug release before colon arrival would become very low. Moreover, due to the relatively long transit time in the ascending colon, release would be likely to occur in an anatomical region still suitable for both dispersion/dissolution of the active ingredient and possible absorption. The prevalent trend was to design time-dependent colon delivery systems with lag phases far longer (6–7 h) than the established mean SITT value. The time needed (up to 2 h) for gastroresistant films to completely dissolve in the small bowel should also be taken into consideration [44]. Importantly, there was also confidence in relatively long transit times in the ascending and transverse colon. In this respect, an interesting paper describing a meta-model to predict movement of non-disintegrating single unit dosage forms through the GI tract has recently been published [45]. Transit data obtained by MMM from 73 subjects were considered. Estimated Mean Residence Time (MRT) in the ascending and transverse colon were, respectively, 545 and 135 min for a total of more than 11 h. Despite criticisms and concerns, the important thing is that there are experimental data that demonstrate the ability of these systems to selectively deliver drugs into the colon, and they really seem to work.

## 2. Time-Controlled Colon Drug Delivery Systems

### 2.1. Capsular Devices with Release-Controlling Plugs

**Figure 10:**
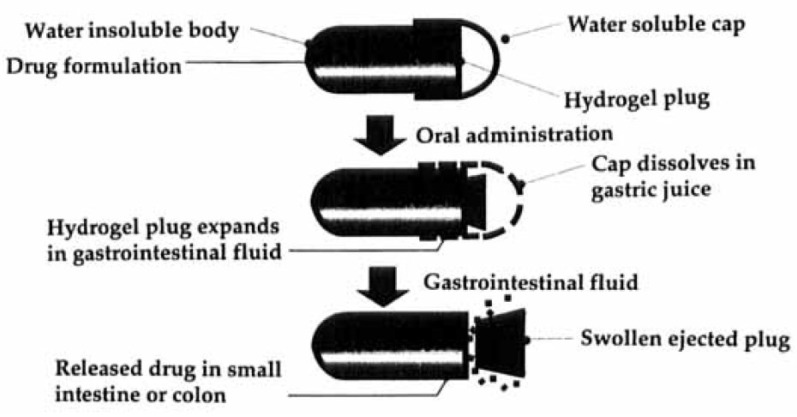
Schematic representation of the Pulsincap™ System. Reprinted with permission from Ref. [47]. 1997, Taylor & Francis.

The Pulsincap^TM^ delivery platform was devised in the form of a capsule having a water-insoluble rigid body, containing the drug formulation, and a sealing hydrogel plug (Figure 10) [33,46,47]. The water-soluble cap was provided with an enteric film. After oral administration, the enteric film and the underlying cap would dissolve in biological fluids when the capsular device leaves the stomach. So, the hydrogel plug starts swelling upon interaction with the aqueous medium. After a lag phase, dependent on the polymer characteristics, the plug thickness and its position inside the capsule body, the swollen hydrogel matrix would be ejected, and a rapid release of the active ingredient would occur. The Pulsincap^TM^ system in this enteric-coated configuration was tested in six fasted volunteers by γ-scintigraphy, and the ejection of the plug was observed in the ascending colon (Table 2).

Notably, SITT was in agreement with the above-mentioned data and the relevant variability was low. Despite the smart design, such a technology suffered from scale-up issues and regulatory constraints associated with the plug-forming hydrogel that was not approved for human use.

**Figure 11:**
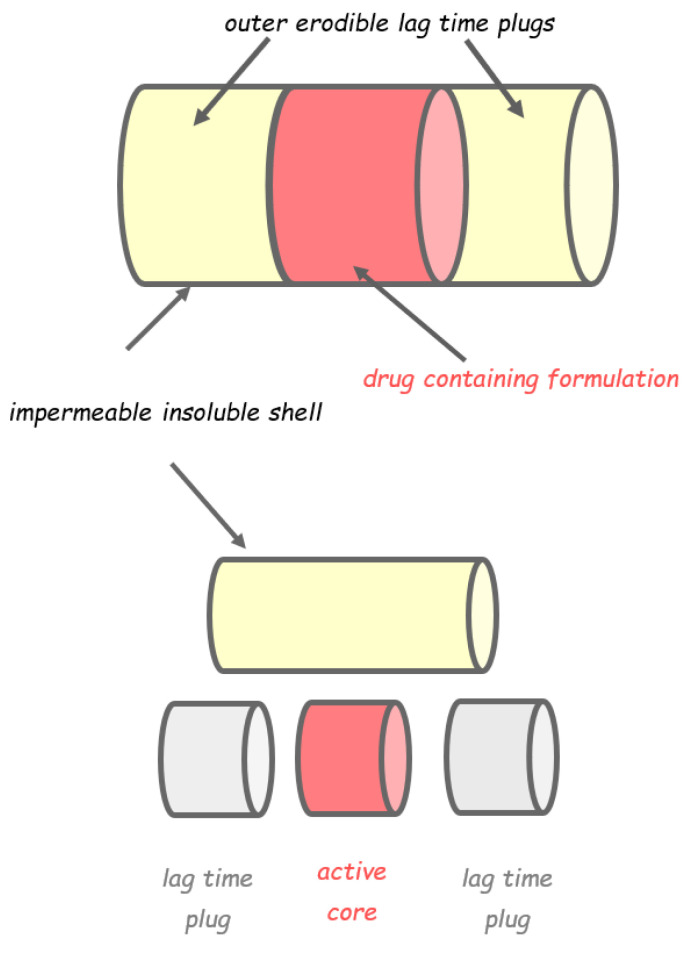
Schematic representation of the Egalet^®^ system. Adapted from Ref. [49].

Another capsular device, the Egalet^®^, consisted of an impermeable cylindrical shell fabricated by injection-molding, containing the drug, and two erodible plugs composed of high-molecular weight polyethylene glycol or polyethylene oxide monostearate and hydroxypropyl methyl cellulose (HPMC) phthalate (Figure 11) [36,48,49]. After oral administration, the plugs would interact with biological fluids and undergo dissolution/surface erosion upon entering the small intestine. When the plugs are completely dissolved, the inner formulation is exposed to the aqueous fluids and the drug is released after a lag period. The delay time imparted by the plugs is dependent on their size and composition. Also in this case, the site of drug release was the ascending or transverse colon in all six subjects (Table 3) [49].

### 2.2. Reservoir Devices with Release-Controlling Coatings

**Figure 12:**
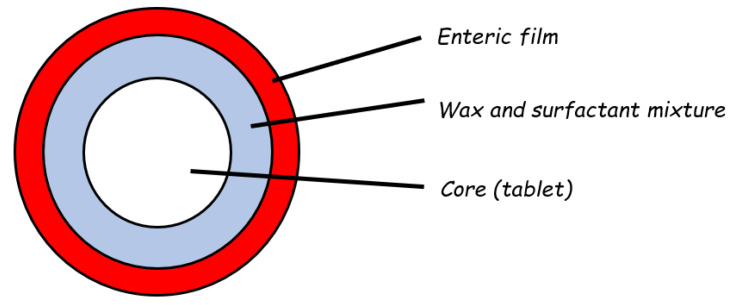
Schematic representation of the Time Clock^®^ system. Adapted with permission from Ref. [50]. 1994, Elsevier.

Reservoir devices are the most common type of time-based formulation for colon delivery. Among these, the Time Clock^®^ system was devised as a tablet core surrounded by an inner layer based on a mixture of natural waxes (carnauba and white beeswax) and surfactant (polyoxyethylene sorbitan mono-oleate), applied by spray-coating at high operating temperatures, and by an outer enteric coating (Figure 12) [34,50,51,52]. The latter film would dissolve in the small intestine, and erosion/dispersion of the waxy layer would then start. After a lag phase of predetermined duration, depending on the thickness of the inner coating, drug release would occur. When tested for release in six fed volunteers, the disintegration of the tablet was consistently seen in the colon (Table 4) [51].

**Figure 13:**
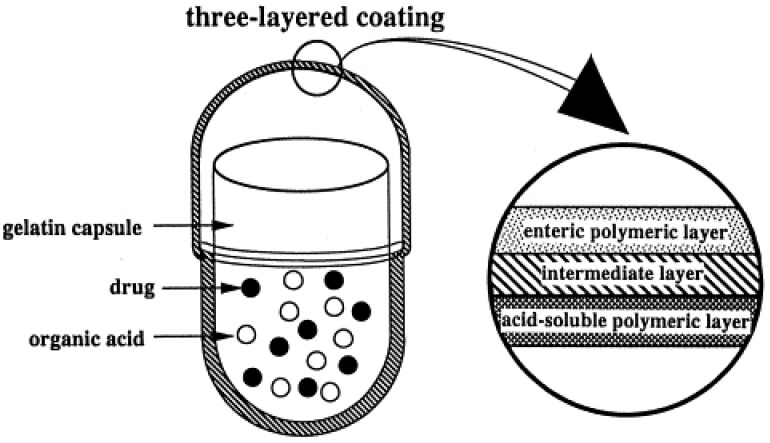
Schematic representation of the CTDC system. Reprinted with permission from Ref. [53]. 1998, Elsevier.

The CTDC system was a reservoir device including a gelatin capsule filled with a mixture of drug with an organic acid, an inner acid-soluble permeable layer (Eudragit^®^ E), an outer enteric coating (hydroxypropyl methyl cellulose acetate succinate, HPMCAS) and a separation layer (HPMC of low viscosity grade) in between (Figure 13) [35,53,54,55]. When administered orally, the enteric and the hydrophilic films would dissolve in the intestine when the dissolution pH threshold of HPMCAS is exceeded, and fluids would diffuse into the capsule through the permeable Eudragit^®^ E layer. As a result, the organic acid dissolves and the low pH of the internal environment promotes progressive dissolution of the acid-soluble coating leading to drug release from the capsule. The lag phase duration depends on the thickness of such a layer. A *γ*-scintigraphy study confirmed the colon targeting ability of this delivery system. Indeed, disintegration of the capsules generally started in the ascending colon and was in all cases completed within the large bowel (Table 5) [55].

**Figure 14:**
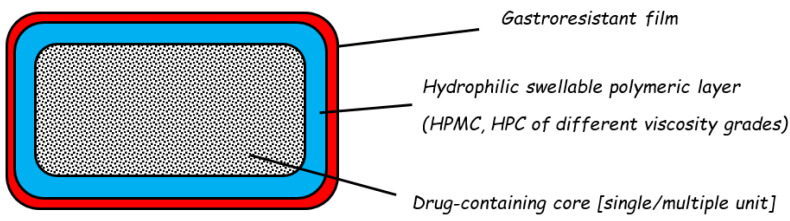
Schematic representation of the Chronotopic^®^ system. Adapted with permission from Ref. [58]. 2009, John Wiley & Sons.

The Chronotopic^®^ system is based on a swellable hydrophilic polymer layer (HPMC or hydroxypropyl cellulose, HPC, of different viscosity grades), responsible for deferring the onset of release, which was applied to drug-containing cores of various nature (single or multiple units) (Figure 14) [1,56,57,58]. To overcome the variability of gastric residence time, an outer enteric film was applied. After swallowing, the enteric film is expected to resist as long as the unit remains in the acidic environment, and to undergo dissolution upon stomach emptying. The inner hydrophilic layer is then exposed to the aqueous medium and a gel would be formed following glass–rubber transition of the polymer. The gel layer would become progressively permeable and/or erode, thus delaying contact between the core and the aqueous fluids. The lag phase duration would vary as a function of the physico-chemical properties of the hydrophilic coating agent and the relevant coating level. Finally, the drug would be released in an immediate or slow mode according to the core characteristics.

**Figure 15:**
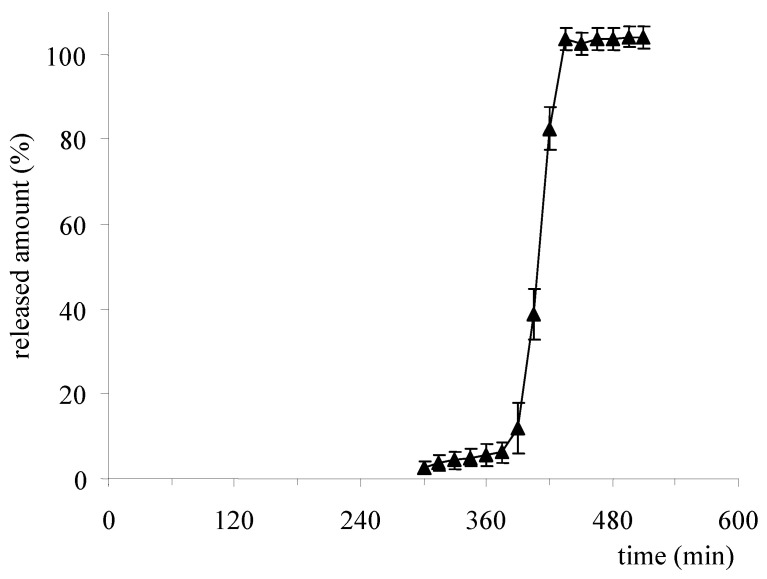
Release profile of verapamil from a Chronotopic^®^ press-coated system having 150% weight gain. Reprinted with permission from Ref. [58]. 2009, John Wiley & Sons.

**Figure 16:**
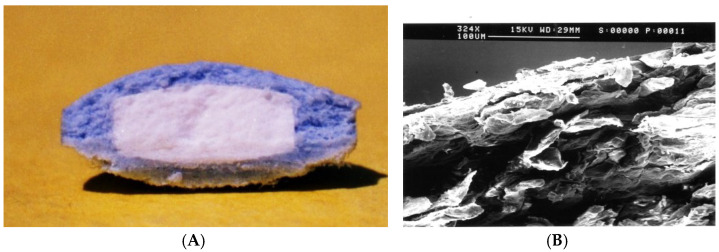
Cross-sectioned press-coated Chronotopic^®^ system (**A**) and SEM photomicrograph of the coating applied (**B**). Reprinted with permission from Ref. [58]. 2009, John Wiley & Sons.

The manufacturing of the Chronotopic^®^ system posed technological challenges mainly related to the coating technique to be used for application of the hydrophilic polymer layer. To this end, press-coating and spray-coating were explored. The former technique was in principle preferred because of a long-standing expertise in the field, also including multi-layer tablets. In vitro release profiles from press-coated systems based on low-viscosity HPMC (Methocel^®^ K100LV) showed reproducible lag times, although a relatively long undesired diffusion phase was observed before (Figure 15) [58]. However, this technique requires special presses for large-scale production and involves difficulties in centering the tablet to be coated within the polymer powder bed, with possible repercussions on consistency of the coating thickness (Figure 16). Moreover, it poses limitations in the design flexibility owing to the large amount of coating polymer needed.

**Figure 17:**
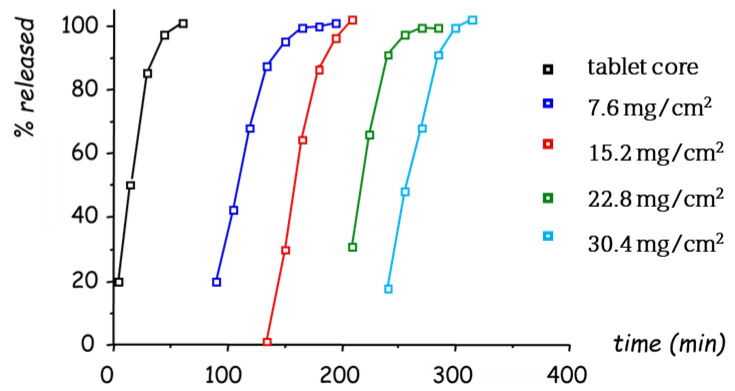
Release profiles of indomethacin from uncoated tablets (4 mm diameter) and systems spray-coated with Methocel^®^ K15M to increasing coating levels. Adapted with permission from Ref. [58]. 2009, John Wiley & Sons.

Thus, the feasibility of spray-coating was explored [57,58,59]. Medium- and high-viscosity HPMC grades had never been used before as coating agents. For this reason, technical issues to achieve acceptable sprayability and reasonable processing time needed to be addressed. To counteract the viscosity-building effect of such polymers, they were used as hydroalcoholic dispersions. From systems coated with a Methocel^®^ K15M ethanol/water dispersion in a rotating pan, the lag time prior to in vitro release was in good agreement with the coating level (Figure 17). However, such a manufacturing technique was poorly fit for large-scale production due to the use of organic solvents.

**Figure 18:**
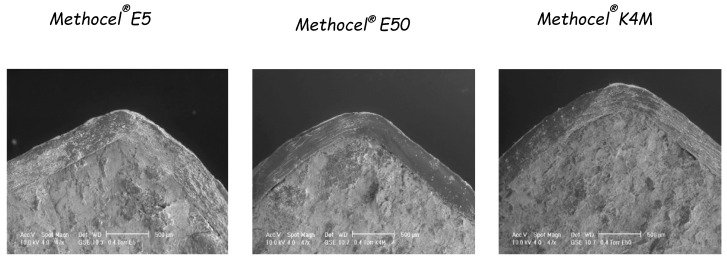
SEM photomicrographs of cross-sectioned systems spray-coated with Methocel^®^ E5, E50 and K4M, respectively, to 20% weight gain. Adapted with permission from Ref. [60]. 2004, Elsevier.

**Figure 19:**
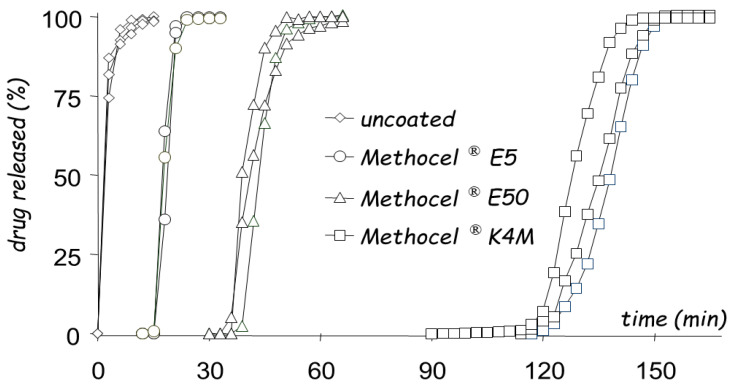
Paracetamol release profiles from uncoated tablets and systems spray-coated with Methocel^®^ E5, E50 and K4M to 20% weight gain. Reprinted with permission from Ref. [60]. 2004, Elsevier.

**Figure 20:**
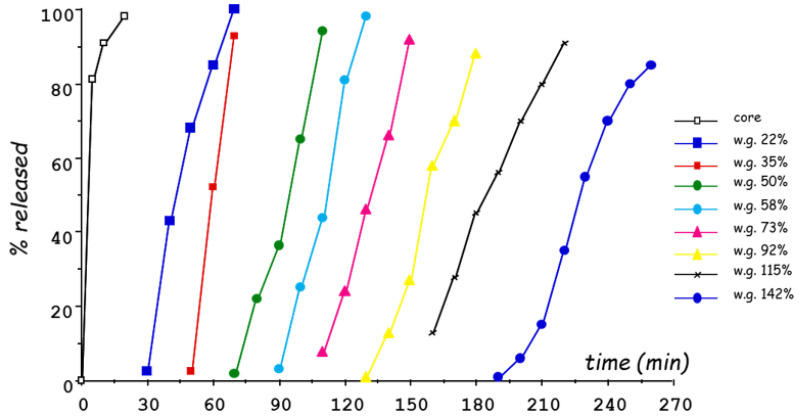
Release profiles of a tracer substance from uncoated tablets and systems spray-coated with Methocel^®^ E50 to increasing weight gains. Adapted with permission from Ref. [58]. 2009, John Wiley & Sons.

**Figure 21:**
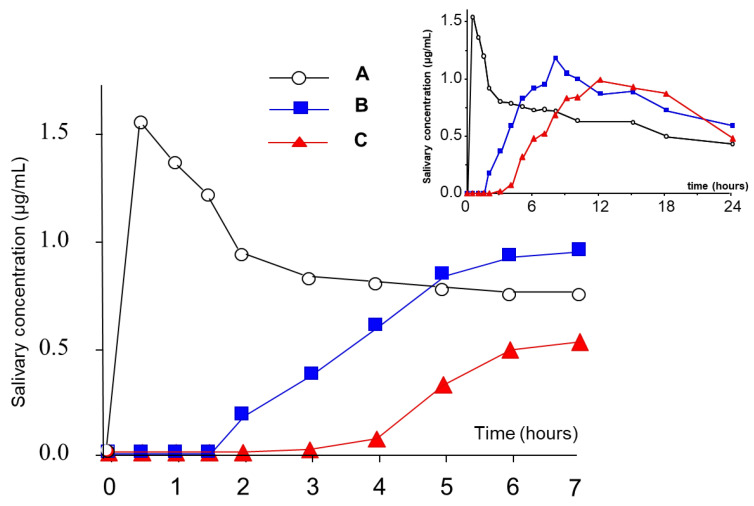
Mean salivary antipyrine concentration profiles after oral administration of uncoated cores (A) and systems coated with Methocel^®^ E50 up to 50% (B) and 100% (C) weight gains. Adapted with permission from Ref. [61]. 2001, Elsevier.

**Figure 22:**
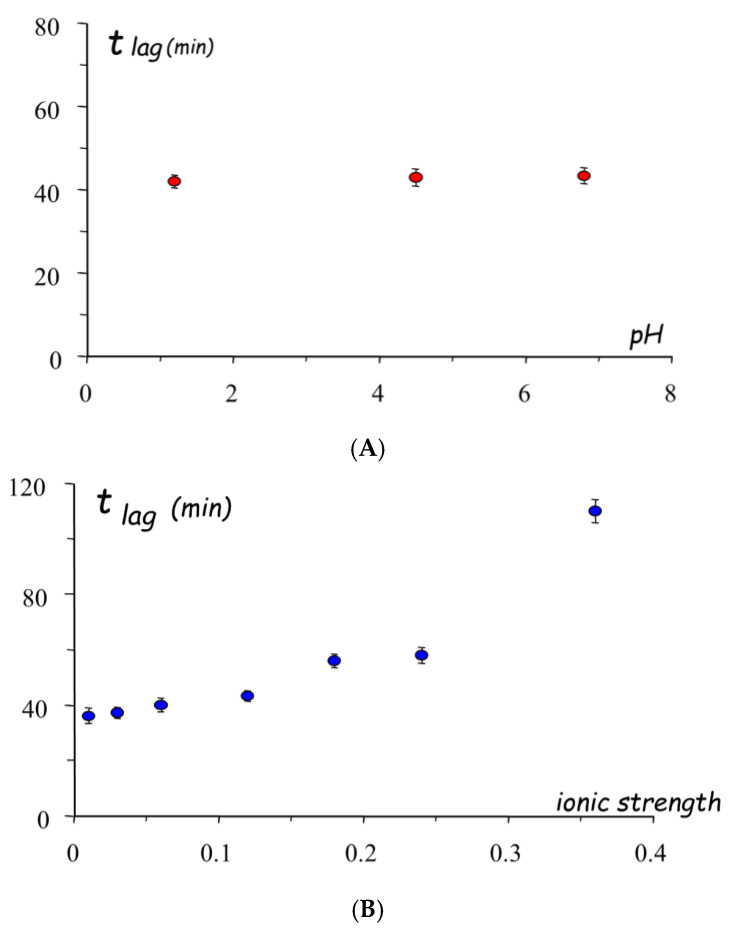
In vitro lag times in media having different pH (**A**) and ionic strength (**B**) of systems coated with Methocel^®^ E50 to 20% weight gain. Adapted with permission from Ref. [60]. 2004, Elsevier.

For these reasons, the use of aqueous HPMC solutions was attempted, and a comparative study was performed to select the most convenient HPMC grades to be employed as coating agents [60]. In particular, aqueous spray-coating was proved feasible with Methocel^®^ E5, Methocel^®^ E50 and Methocel^®^ K4M (Figure 18). The release profiles obtained from systems having coatings of 300 µm in nominal thickness showed increasing lag times as a function of the HPMC viscosity grade (Figure 19). A longer delay phase was obtained from Methocel^®^ K4M-based formulations. However, Methocel^®^ E50-coated systems showed better results in terms of in vivo performance, coating process time, process feasibility and fine tuning of the lag phase (Figure 20 and Figure 21) [58,60,61]. Indeed, a good correlation was found between weight gain and lag time. When in vitro testing was carried out in media having different pH (1.2–6.8) and ionic strength (0.01–0.60) values, consistent lag phases were achieved regardless of such variables in their physiological ranges (Figure 22). Moreover, the coating process was shown to be robust and potentially scalable. In the case of Methocel^®^ K4M, it was hampered by the high viscosity of its water solutions. From Methocel^®^ K4M-coated units, a small percentage of the model drug was slowly released toward the end of the delay period, which was also reflected in human salivary concentration profiles [62]. This behavior was attributed to the formation of a poorly erodible gel structure rupturing upon water inflow and consequent disintegration of the tablet core.

**Figure 23:**
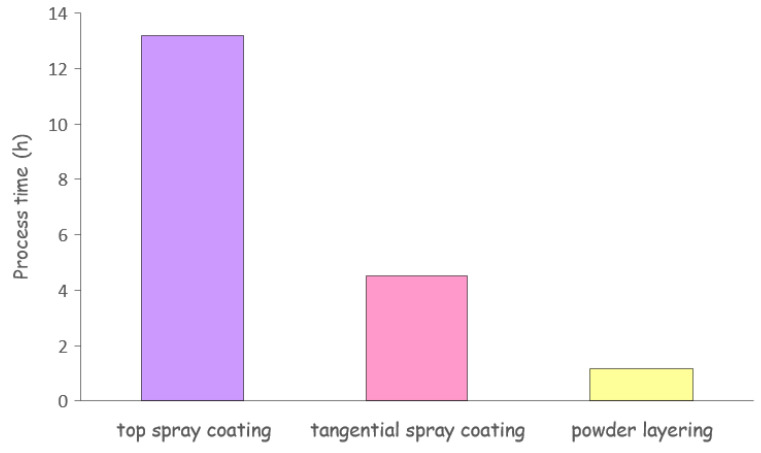
Process times for application of Methocel^®^ E50 to 50% weight gain by different coating techniques.

In order to overcome the technical issues related to the long process time required for aqueous spray-coating, different techniques and equipment were evaluated [63,64]. Namely, top spray- and tangential spray-coating as well as powder-layering were explored. Among these techniques, top spray-coating was shown to be more time-consuming, while tangential spray-coating, used to apply HPMC (Methocel^®^ E50) in aqueous solution or powder, required much shorter process times (Figure 23). Indeed, tangential spray-coating and powder-layering process times to achieve 50% weight gain were reduced by approximately 30% and 10% as compared to top spray-coating, respectively.

**Figure 24:**
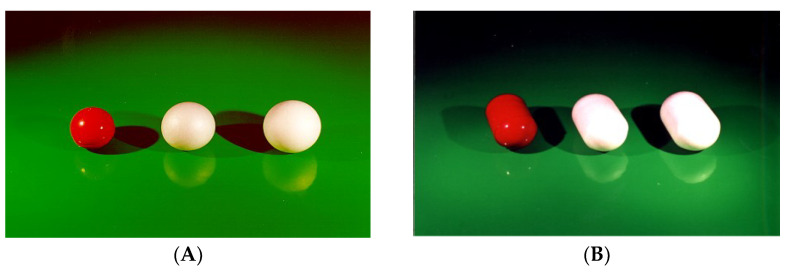
Chronotopic^®^ systems having soft-gelatin (format 2 round) (**A**) and hard-gelatin capsules (DBcaps, size B) (**B**) as the core.

**Figure 25:**
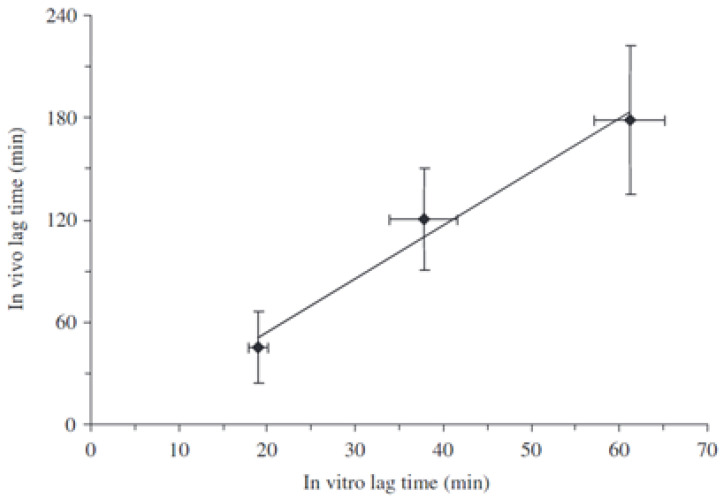
Relationship between in vitro t_10%_ (time to 10% release) and in vivo t_10%_ (time to 10% C_max_) for hard-gelatin capsule-based systems coated with Methocel^®^ E50 to increasing HPMC layer thicknesses. Reprinted with permission from Ref. [58]. 2009, John Wiley & Sons.

The Chronotopic^®^ technology has successfully been applied to various solid dosage forms, i.e., tablets of different sizes, hard- and soft-gelatin capsules, pellets and minitablets [58]. Although challenging, the use of capsule cores was investigated in-depth because of many related advantages, including the possibility of incorporating liquid, semisolid and multiparticulate formulations such as emulsions, microemulsions, self-microemulsifying drug delivery systems, solid lipid nanospheres, microparticle suspensions and pro-liposomes (Figure 24). In vivo results obtained from HPMC-coated hard-gelatin capsules type DBcaps^®^ size B confirmed the performance already observed with tablet cores. When in vitro and in vivo t_10%_ data obtained from HPMC-coated capsules were matched, a good correlation was found (Figure 25).

**Figure 26:**
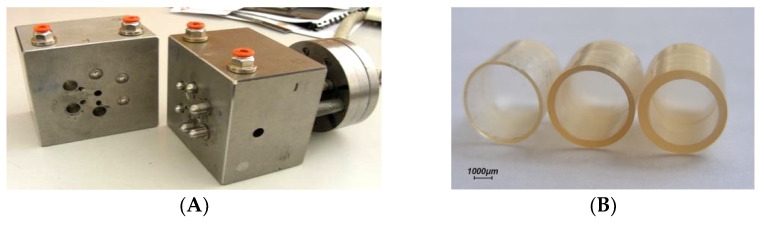
Photographs of the mold set for fabrication of Chronocap™ capsule shells by injection-molding (**A**) and capsules bodies based on HPC (Klucel^®^ LF) having 300, 600 and 900 µm thickness (**B**). Adapted with permission from Ref. [67].

**Figure 27:**
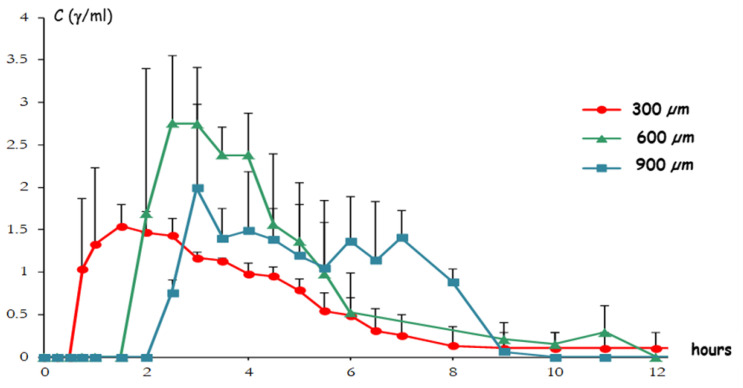
Mean salivary paracetamol concentration profiles after oral administration of Klucel^®^ LF-based Chronocap^TM^ systems having different thicknesses. Bars represent standard deviation. Adapted with permission from Ref. [67].

**Figure 28:**
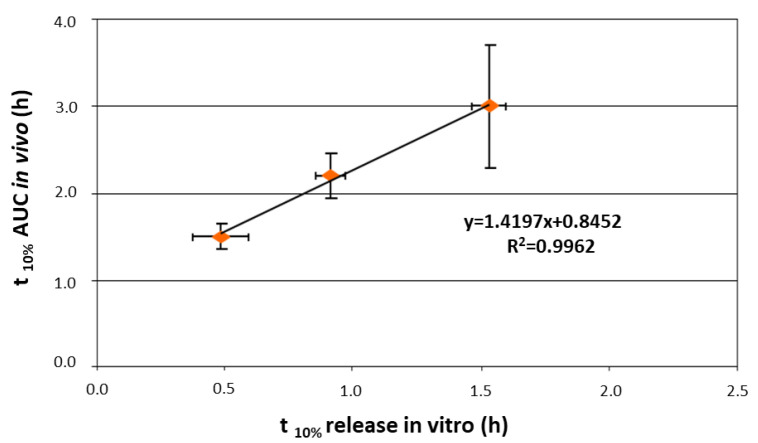
Relationship between in vitro t_10%_ (time to 10% release) and in vivo t_10%_ (time to 10% AUC) for Klucel^®^ LF-based Chronocap^TM^ systems having increasing thicknesses. Adapted with permission from Ref. [67].

A new delivery platform (Chronocap™) was subsequently developed, which combined the above-mentioned advantages related to coated capsules and the release-modifying properties of hydrophilic cellulose derivatives. Such a system was devised in the form of capsular shells having caps and bodies intended for assembly after filling with drug formulations of various natures. For the manufacturing of such shells, an innovative technique, i.e., injection-molding was exploited [65,66]. Interestingly, independent pharmaceutical development of the inner formulation and the outer shell was allowed. The functional capsules were manufactured from HPC of different viscosity grades, plasticized with polyethylene glycol 1500. The polymer mixture was loaded into a bench-top micromolding machine, equipped with capsule-shaped molds for caps and bodies. Shells having different thicknesses were obtained, namely 300, 600 and 900 µm (Figure 26). In vivo salivary concentration profiles of a model drug showed longer delay phases as a function of the thickness of the shell, as desired, and a good in vitro–in vivo correlation was found between in vitro t_10%_ and in vivo t_10%_, the latter expressed as the time to 10% of AUC (Figure 27 and Figure 28) [67].

**Figure 29:**
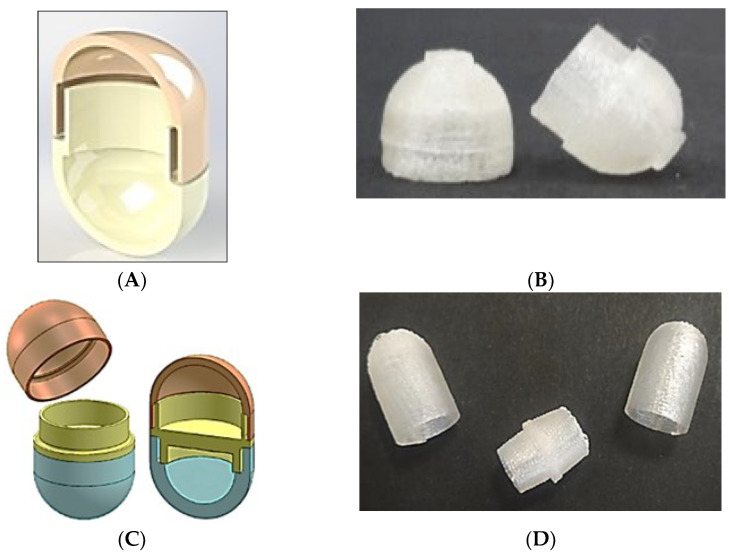
CAD designs of a cross-sectioned capsule (**A**) and multicompartment capsular system (**B**), and photographs of 3D printed body and cap (**C**) and bodies and spacer (**D**) fabricated by FDM 3D printing from Klucel^®^ LF. Adapted with permission from Ref. [70]. 2017, Elsevier.

The functional capsules were subsequently replicated by Fused Deposition Modeling (FDM) 3D printing, from HPC filaments fabricated in-house by hot-melt extrusion [68,69]. The shell parts were fabricated based on Computer-Aided Design (CAD) files purposely developed, after assessing the possibility of attaining hollow structures by the use of FDM, which had not been demonstrated previously (Figure 29). The in vitro release profiles of the printed capsules fully corresponded to those exhibited by molded capsules having the same nominal thickness. These results would support the real-time prototyping potential of FDM *vs.* injection-molding technique, more advantageous in terms of processing time and scalability for the manufacturing of the functional shells. The experience acquired in the use of FDM led to the fabrication of systems of more complex design, consisting of caps having different thicknesses and/or compositions assembled through one or more joints that would also serve as a partition [70]. The resulting multi-compartment devices would offer versatile release performance, such as successive release pulses, that could well meet the needs related to personalization of the therapy.

**Figure 30:**
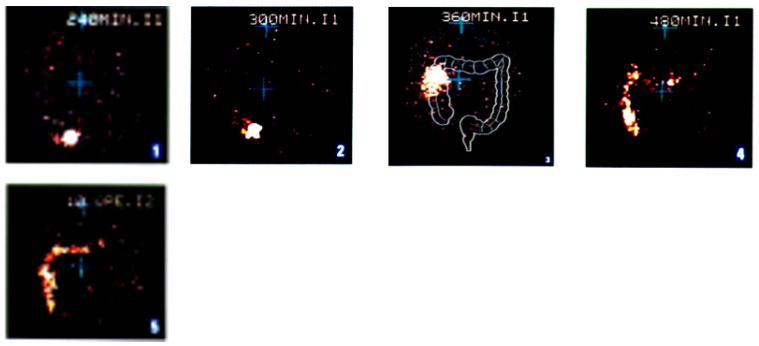
*γ*-scintigraphy of double-coated *placebo* Chronotopic^®^ systems (Methocel^®^ E50 applied to 100% weight gain) relevant to subject #6.

All the HPMC coated units (tablets, pellets, gelatin capsules) and HPC capsular devices fabricated by injection-molding and 3D printing provided with an outer gastroresistant film could act as time-controlled colon drug delivery systems. In particular, the fate of samarium oxide-labeled *placebo* (tablet core 6 mm, 160 mg) coated with Methocel^®^ E50 (100% weight gain, ≈ 900 µm thickness) and Eudragit^®^ L in six fasted healthy male volunteers was studied by *γ*-scintigraphy (Table 6) [61]. In all cases, the units were seen to disintegrate in the ascending colon (Figure 30).

**Figure 31:**
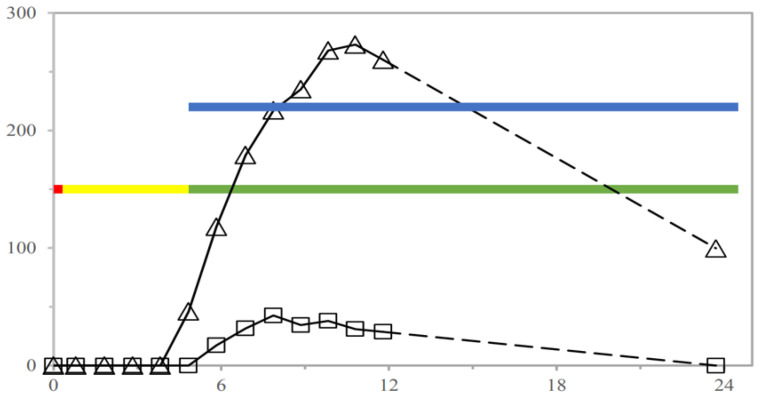
5-ASA (□) and N-acetyl 5-ASA (Δ) plasma concentration profiles following administration of double-coated Chronotopic^®^ systems (Methocel^®^ E50 applied to 50% weight gain) to one fasted volunteer (subject #2). The dashed portion of the curves indicates the 12–24 h time frame during which no experimental data were collected and does not reflect the actual time course of concentration. Red, yellow and green bars (bottom) indicate gastric, small intestinal and colonic residence, respectively; the blue bar (top) indicates disintegration. Reprinted with permission from Ref. [71]. 2019, Elsevier.

**Figure 32:**
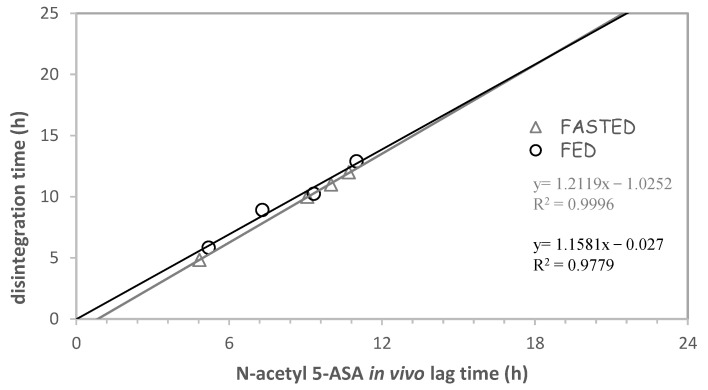
Relationship between N-acetyl 5-ASA in vivo lag time and time of disintegration of double-coated Chronotopic^®^ systems (Methocel^®^ E50 applied to 50% weight gain). Adapted with permission from Ref. [71]. 2019, Elsevier.

A subsequent pharmaco-scintigraphy investigation was carried out using 5-ASA systems in both fasted and fed volunteers according to a randomized two-period crossover design [71]. A lag phase preceded appearance of the drug and the metabolite in the plasma (Figure 31). As expected, 5-ASA levels turned out to be far lower than N-acetyl 5-ASA, resulting from intestinal and hepatic metabolism of the parent drug. Their concentrations would indicate poor absorption, possibly consistent with distal intestinal release. The drug and metabolite were detected in the plasma in conjunction with disintegration of the unit, which occurred in the large bowel. In 8 out of 12 cases, breakup of the units occurred into the ascending or transverse colon (Table 7). A linear correlation was found between N-acetyl 5-ASA in vivo lag time and time of disintegration of the systems irrespective of the fasted or fed state (Figure 32).

**Figure 33:**
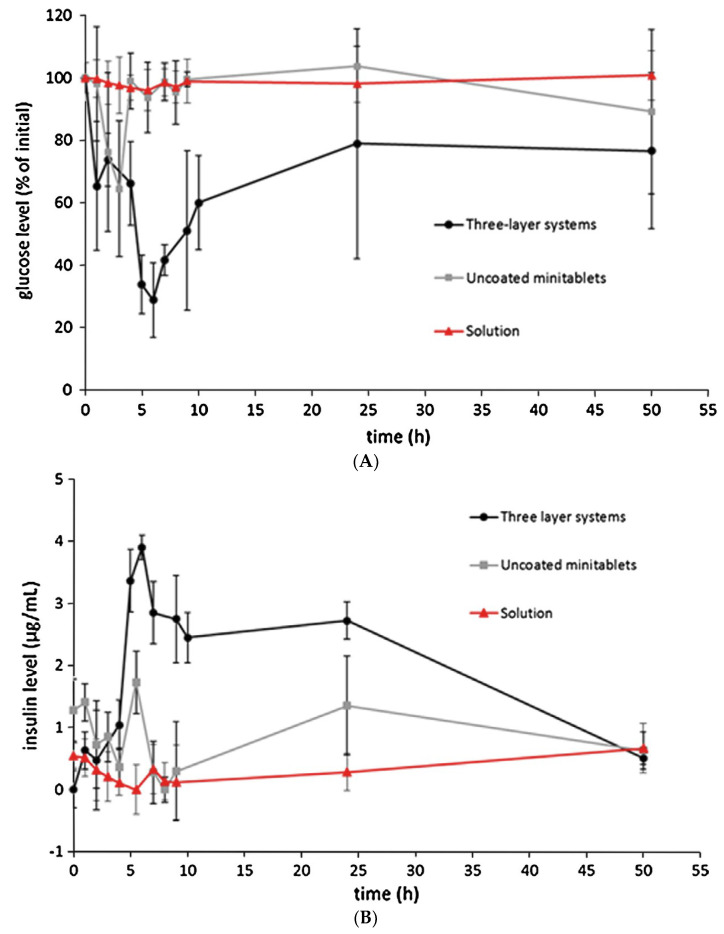
Plasma glucose (**A**) and insulin (**B**) concentration profiles in diabetic rats following oral administration of minitablet-based Chronotopic^®^ systems, uncoated minitablets or insulin in solution (bars indicate standard deviation). Reprinted with permission from Ref. [74]. 2016, Elsevier.

A multiple-unit Chronotopic^®^ system, having minitablets as the core and improved efficiency in deferring the onset of release, was obtained by applying an additional permeable film based on neutral polymethacrylate Eudragit^®^ NE and superdisintegrant sodium starch glycolate onto the HPMC layer [72,73]. Results from three-layer insulin systems confirmed the colon targeting reliability of the delivery platform in in diabetic rats also (Figure 33) [74,75]. Although it has been reported that the human GI transit may hardly be predicted by the use of animal models, the rat has interestingly been considered as one of the most reliable tools in this respect [76,77].

## 3. Conclusions

Colon delivery of bioactive compounds administered orally has become, despite the poor absorption properties of this intestinal region, an important topic of pharmaceutical research in recent decades. For all proposed strategies, the development of oral colon delivery systems involves unique, hard to face challenges. In fact, biological variability can heavily influence the peculiar physiological parameters underlying the different approaches. This may be especially true under pathological conditions. For colon targeting, various drug delivery platforms have been proposed, including time-dependent systems that rely on relatively constant transit time along the small intestine. Systems based on small intestinal transit time are able to control the onset of release, which is expected to occur after a programmed delay time in order to cover the entire transit from the duodenum to the ileo-cecal junction. The desired lag phase is mainly pursued through the use of polymeric coatings or capsule plugs, and enteric coating is needed to overcome the influence of the gastric emptying variability. Particularly challenging is the development of in vitro testing methods to assess consistency of the lag phase duration. Experience has shown that it is possible to set up drug release tests sensitive enough to reflect specific formulation interventions. However, it is essential to collect a robust set of human in vivo data in order to establish useful in vivo–in vitro associations. The results of *γ*-scintigraphy and pharmaco–scintigraphy studies collected from the main oral delivery platforms for time-dependent colon targeting presented and discussed in this review article demonstrate that this approach has led to a satisfactory outcome in the great majority of cases, thus indicating the considerable potential available and laying the base for further and broader exploitation.

## Figures and Tables

**Table 1:** Patents of time-controlled delivery platforms proposed for colonic release, for which proof-of-concept was achieved by human *γ*-scintigraphy studies.

Delivery Platform	Type	Patent Priority Date	Patent n.
Chronotopic^®^	Tablet device	20 October 1988	US 5,171,580 [32]
Pulsincap™	Capsule device	16 February 1989	WO 90/09168 [33]
Time Clock^®^	Tablet device	4 July 1990	GB 2,245,492 [34]
CTDC	Capsule device	20 July 1995	US 6,309,666B1 [35]
Egalet^®^	Cylindrical container device	3 April 1997	CA 2,327,685 [36]

**Table 2:** Transit and plug separation times (h) of *placebo* units in six fasted volunteers. Adapted with permission from Ref. [47]. 1997, Taylor & Francis.

Subject	Gastric Residence	Small Intestine Transit	Colon Arrival	Plug Separation Post-Dose	Plug Separation Post-GE	Ascending Colon Residence
1	0.19	3.31	3.50	3.68	3.49	6.33
2	0.56	3.38	3.94	4.52	3.96	5.14
3	0.86	2.77	3.63	5.07	4.21	7.73
4	0.27	3.33	3.60	4.13	3.86	2.63
5	0.81	3.07	3.88	4.50	3.69	6.03
6	0.26	3.32	3.58	10.48 ^1^	10.22 ^1^	8.20
Mean	0.49	3.20	3.69	5.40	4.91	6.01
SD	0.30	0.24	0.18	2.53	2.62	2.00

^1^ estimated and not considered in the mean calculation.

**Table 3:** Gastric residence (h), release time (h) and release site of labeled units in six fasted volunteers. Adapted from Ref. [49].

Subject	Gastric Residence	Release Time	Release Site
1	1.00	5.67	Ascending colon
2	0.67	6.33	Ascending colon
3	0.33	7.00	Transverse colon
4	1.67	8.00	Transverse colon
5	0.67	5.67	Ascending colon
6	0.67	8.00	Ascending colon
Mean	0.84	6.78	
SD	0.46	1.07	

**Table 4:** GI transit times (min), tablet dispersion time (min) and tablet dispersion site of *placebo* units—six fed (light breakfast) volunteers. Adapted with permission from Ref. [51]. 1994, Elsevier.

Subject	Gastric Residence	Small Intestine Transit	Colon Arrival	Tablet Dispersion	Position of Dispersion
1	103	248	351	655	Cecum
2	251	168	419	656	Proximal colon
3	154	267	421	655	Cecum
4	123	186	319	593	Proximal colon
5	87	163	250	523	Descending colon
6	201	251	452	575	Proximal colon
Mean	153	261	369	610	
SE	27	19	31	23	

**Table 5:** CTDC transit and disintegration times of *placebo* units—eight fasted volunteers. Adapted with permission from Ref. [55]. 1998, Elsevier.

Subject	Initial Disintegration			Complete Disintegration	
	Minutes Post-Dose	Minutes Post-GE	Anatomical Position	Minutes Post-Dose	Anatomical Position
1	371	324	Ileo-cecal junction	422	Ascending colon
2	310	282	Ascending colon	421	Ascending colon
3	304	241	Ileo-cecal junction	514	Ascending colon
4	298	272	Descending colon	469	Descending colon
5	385	349	Ascending colon	495	Ascending colon
6	663	590	Ascending colon	685	Ascending colon
7	240	201	Ascending colon	301	Ascending colon
8	283	270	Ascending colon	502	Transverse colon
Mean	357	316		476	
SD	132	120		109	

**Table 6:** Transit and disintegration times (h) of double-coated Chronotopic^®^ systems (Methocel^®^ E50 applied to 100% weight gain) in six volunteers. Data acquired by *γ*-scintigraphy. Adapted with permission from Ref. [61]. 2001, Elsevier.

Subject	Gastric Residence	Small Intestine Transit	Colon Arrival	Breakup Time after Gastric Emptying	Breakup Site
1	1.0	7.0	8.0	7.0	Cecum/ascending colon
2	2.0	5.0	7.0	6.0	Ascending colon
3	0.5	3.5	4.0	4.5	Cecum/ascending colon
4	0.5	4.5	5.0	5.5	Ascending colon
5	1.0	4.5	5.5	5.0	Cecum/ascending colon
6	0.5	4.5	6.0	6.0	Ascending colon
Mean	0.9	5.0	5.9	5.7	
SD	0.5	1.1	1.3	0.8	

**Table 7:** Transit and disintegration times (h) of double-coated 5-ASA Chronotopic^®^ systems (Methocel^®^ E50 applied to 50% weight gain) under fasted and fed conditions. Adapted with permission from Ref. [71]. 2019, Elsevier.

**Fasted**					
**Subject**	**Gastric** **Residence**	**Small** **Intestine Transit**	**Colon** **Arrival**	**Breakup Time after Gastric Emptying**	**Breakup Site**
1	1.75	3.00	4.75	10.25	Transverse colon
2	0.33	4.50	4.84	4.50	Cecum
3	0.42	1.50	1.92	10.58	Ascending colon
4	0.42	4.58	5.00	9.58	Ascending colon
5	0.58	4.50	5.08	10.52 ^1^	Transverse colon
6	0.58	1.59	2.17	12.59	Transverse colon
Mean	0.68	3.28	3.96	8.73	
SD	0.53	1.47	1.49	2.85	
**Fed**					
**Subject**	**Gastric** **Residence**	**Small** **Intestine Transit**	**Colon** **Arrival**	**Breakup Time after Gastric Emptying**	**Breakup Site**
1	1.83	1.92	3.75	-	-
2	2.33	2.50	4.83	3.50	Ascending colon
3	1.41	3.51	4.92	7.51	Ascending colon
4	2.50	2.50	5.00	10.40	Ascending colon
5	1.92	3.00	4.92	11.95 ^1^	Ascending colon
6	0.58	3.59	4.17	9.67	Transverse colon
Mean	1.76	2.84	4.60	7.77	
SD	0.70	0.65	0.51	3.10	

^1^ estimated and not considered in the mean calculation.

## Data Availability

Not applicable.
